# An evaluation of rheumatology practitioner outreach clinics: a qualitative study

**DOI:** 10.1186/1472-6963-12-119

**Published:** 2012-05-20

**Authors:** Asmaa S Abdelhamid, Janice Mooney, Andrew A Walker, Garry Barton, Alex J MacGregor, David GI Scott, Richard A Watts

**Affiliations:** 1 Norwich Medical School, University of East Anglia, NR4 7TJ, Norwich, Norfolk, UK; 2 School of Nursing Sciences, University Of East Anglia, Anglia, UK; 3 Clinical Research and Trials Unit, Norfolk and Norwich University Foundation NHS Trust, Norfolk, Norwich, UK; 4 Department of Rheumatology, Norfolk and Norwich University Foundation NHS Trust, Norfolk, Norwich, UK

## Abstract

**Background:**

Services for Rheumatoid Arthritis (RA) have evolved with the development of independently led outreach Rheumatology Practitioner (RP) clinics in Primary Care (PC). Their clinical and cost effectiveness, compared with Secondary Care (SC) services, has not been assessed. The RECIPROCATE study aims to evaluate their clinical and cost effectiveness. This part of the study aimed to explore health professionals’ opinions of rheumatology outreach service.

**Methods:**

Using a qualitative design, semi-structured interviews were conducted with GPs, practice nurses, hospital doctors and RPs, from one hospital and seven PC practices in Norfolk, to elicit their opinions of the service. The interviews were analysed using thematic analysis.

**Results:**

All participants agreed the service was supportive and valuable providing high quality personalised care, disease management, social, and educational support. Advantages identified included convenience, continuity of care and proximity of services to home. RPs helped bridge the communication gap between PC and SC. Some participants suggested having a doctor alongside RPs. The service was considered to be cost effective for patients but there was uncertainty about cost effectiveness for service providers. Few disadvantages were identified the most recurring being the lack of other onsite services when needed. It was noted that more services could be provided by RPs such as prescribing and joint injections as well as playing a more active role in knowledge transfer to PC.

**Conclusions:**

Professionals involved in the care of RA patients recognised the valuable role of the RP outreach clinics. This service can be further developed in rheumatology and the example can be replicated for other chronic conditions.

## Background

Rheumatoid arthritis (RA) is the commonest chronic debilitating, destructive inflammatory arthritis affecting 0.8% of the population [1]. RA is a multisystem disease that can affect the lungs, skin, heart and eyes and is associated with an increased risk of cardiovascular disease equivalent to that seen in type 2 diabetes [2]. The British Society for Rheumatology (BSR) and European League Against Rheumatism (EULAR) guidelines for the management of RA recommend early diagnosis and treatment using disease modifying anti-rheumatic drugs (DMARDs) or biologic agents [3,4] . Optimal disease management requires early initiation of DMARDs and escalation of therapy to reach therapeutic dosages in order to achieve control of inflammation. Long-term treatment with immunosuppressive agents is necessary; hence patients require regular monitoring to assess disease activity and progression, provide early detection of drug toxicity, and manage co-morbidities.

The delivery of care for RA patients has changed over the past two decades. Rheumatology nurses have become an integral part of the multidisciplinary team. They have expanded their role to include a range of activities involved in the assessment and management of RA such as musculoskeletal examination, medication monitoring , patient education, joint injections, nurse prescribing, refererrals to other health professionals and follow up clinics [5-8].

Current NHS policy calls for chronic disease assessment and management to be delivered by primary care close to the patient's home in the community [9]. In 1995, rheumatology nurse outreach clinics were established in primary care in Norfolk. These services have evolved uniquely to provide traditional hospital based services into seven General Practitioner (GP) practices, which are independent of consultant care [10-12].

RA patients require regular monitoring to assess disease progression and early detection of drug toxicity. Outreach clinics are one means by which specialist services can provide care in the community, however, it is not clear that they are always cost effective. Evidence from studies of doctors from secondary care in outreach suggests that the costs are greater, but that patient satisfaction is high [13,14]. Whether the same is true for nurse practitioners in the community is not known. Specialist outreach services are often viewed as dispensable and easy targets in times of financial stringency. However these services are valued by patients [15].

There has not been a formal evaluation of the benefits of this approach to patients with rheumatoid arthritis. In Norfolk, there is a unique pattern of care with a mixture of care provided both in hospital and the community by nurses and doctors independent of SC. What is not known is whether care provided in the community is as a good as conventional hospital based care in terms of clinical benefits, costs, continuity and accessibility for patients. As part of a detailed appraisal of rheumatology services provided in primary and secondary care we have completed a qualitative study exploring the opinions of doctors and nurses providing rheumatology services in Primary and Secondary care in Norfolk about these community based services.

## Methods

### Study design

A qualitative design using semi-structured interviews was chosen for this study as it allowed the exploration of health professionals’ views and opinions. It also enabled health professionals involved in the care of RA patients to reflect on and assess the service provided.

### Setting

The study was set in rural area surrounding a central city (Norwich). Rheumatology Practitioner clinics are provided in seven Primary Care practices located in**;** Holt, Wymondham, Thorpewood (Norwich), Sheringham, North Walsham, Fakenham and Attelborough. Secondary care led services are provided at the main Secondary care hospital (the Norfolk and Norwich University Hospital) by a team comprising five consultants, four specialist registrars and 5 nurse practitioners. Each Primary Care centre is linked to one RP. Each practitioner is trained in the assessment of RA, monitoring for drug toxicity and co-morbidities. Any recommended changes in medication are made to the local GP. Patients in need of specialist care, for example assessment for biologic therapies, are referred back to Secondary care.

### Participants

We invited all seven participating practices to nominate one doctor and one practice nurse each. We also invited all five rheumatology practitioners and five rheumatology hospital doctors selected purposively to be representative of all grades and experience to take part in a semi-structured interview.

### Interview

An interview guide was developed by the research team that included clinicians (see Table 1) based on a framework of service issues perceived to be relevant to the research aim. The interview guide was altered to explore emerging themes after a pilot interview. One interviewer (AA) conducted all interviews at the interviewees’ workplace. The interviews were audio recorded and later transcribed verbatim. Interviews lasted between 20 and 45 minutes.

**Table 1:** Interview topics discussed with health professionals

No.	Topic
1	Perception of outreach rheumatology service setting in Norfolk
2	The advantages and challenges of the service for patients compared with hospital service.
3	Services provided currently or should be provided
4	Types of patients, conditions and referral pathways to the service
5	Communication between Primary care outreach and Secondary care
6	Cost effectiveness and quality of care

### Data analysis

Transcripts were coded and analysed using a Framework Analysis approach [16]. Different researchers coded transcripts independently (AA, JM, AW), with good conceptual agreement. This work was used to inform discussion of the emergent codes and thematic framework. We grouped codes together under: basic themes, organising themes and global themes. We actively sought examples that did not fit with emerging codes and re-visited transcripts to refine and validate the findings. The coding frame eventually covered all areas of data, and corresponded to themes included in the interview guide plus some emergent issues.

### Ethical considerations

All participants received detailed information about the study, that participation was voluntary and that they could withdraw from the study without obligation or giving notice. At the interview, all participants gave their written consent to participate. All transcripts were de-identified. The study was approved by Norfolk Research Ethics Committee08/H0310/127.

## Results

Six out of the seven practices with rheumatology outreach clinics agreed to take part in the study. All five RPs and five rheumatology doctors were interviewed. In total 22 interviews were conducted. Due to a technical fault, the recordings from two practices were not transcribable. Eighteen interviews (5 RPs, 4PC Drs, 5 SC Drs (3 consultants and 2 trainees, and 4 practice nurses) in total were transcribed. Transcripts of 18 interviews plus written notes from the technically void four interviews were used in the analysis.

### Perception of service

Outreach rheumatology services were perceived, by almost all interviewees, as supportive and valuable service independently led by specialist rheumatology practitioners:

“They are a huge resource for us….taken a big workload of doctors in hospitals” (Hospital doctor 1)

“We love having the service here. It‘s quick…it‘s marvellous whereas if a patients coming to the Norfolk and Norwich it is at least a few weeks to be seen normally”. (GP1)

The service was described as providing high quality personalised care, providing monitoring of disease for patients with RA or other inflammatory arthritis requiring disease-modifying drugs. It also provides a social, educational and support service for nurses and patients.

“Very useful source of information about what to do with a patient”. (GP4)

‘The biggest complaint we have from patients here is that when they go to a secondary care clinic they see somebody they’ve never seen before, who reads all their notes, goes right back in their history – so they’ve got 10–20 minutes with that person, 15 minutes is taken up going back over their history then 5 minutes saying – well I don’t think you’ve got this actually. And they come away feeling confused… with (RP), she knows them. It’s a quality appointment” (Practice nurse 4)’

“I see my responsibility out there is to provide support, monitoring and education to those patients and their families, and those patients won’t necessarily come into secondary care” (RP3 )

### Current and possible services provided by rheumatology outreach clinics

Current services identified as being provided by rheumatology practitioners included; ordering blood tests for disease monitoring, assessment of disease activity, ordering investigations, providing advice and counselling, and referring to other members of the multidisciplinary team. Although none of the RPs was a qualified prescriber at the time of conducting the study, they communicate with PC and SC doctors to request alteration to dosage of medication based on their assessment of the case. As the RPs work across both PC and SC this was achievable without patients requiring extra appointments.

It was also noted that RPs could provide additional services such as prescribing and joint injections as well as playing more active role in knowledge transfer to primary care professionals. However, there was concern from several GPs working in practices where joint injections are provided that, although helpful and would ease their workload; this may deskill GPs currently providing the service.

“ (RPs) form a management plan and speak to a doctor if they had any queries” (hospital doctor 2)

“monitoring service …link between the community and the hospital so they’ll highlight, they’ll trouble-shoot and come to me or one of my colleagues and say – this is what’s going on, what do you think?” (Hospital doctor 4)

“I think there might be a role, if there was a protocol written with pre-agreed parameters to give people a steroid injection, or to put up a dose of a disease-modifying drug that had already been started so if somebody comes to clinic and their rheumatoid isn’t well-controlled then you can give them a Depo Medrone injection and put their Methotrexate up from 10 mg to 15 mg a week”. (Hospital doctor 3)

“They can offer advice and support to primary care professionals in managing patients with rheumatological diseases” (GP2)

### Perceived advantages for patients

All participants identified numerous advantages. The most commonly recurring were convenience, continuity of care and providing services closer to home. Specifically, continuity of care was considered important as the continuity of RP allowed for the development of relationships built upon experience based knowledge of the participants’ medical and social circumstances and an increased ability to identify changes from the normal behaviour and functioning of the patient.

“More empathy than doctors and they’ll listen to patients” (Hospital doctor1)

“Continuity of care is probably the major one” (GP3)

*‘That person knows their carers, knows their family, will go to a funeral with them. She’s brilliant.’* (Practice nurse 4)"

“RPs are around for a lot longer than a junior doctor, better consistency of care and relationship and that person can assess more quickly, more easily if there has been any deviation from what’s normal for you, and that’s beneficial”.(Hospital doctor3)

The service was perceived as saving patients the time needed to travel to hospital which is particularly important for an elderly population with a mobility limiting illness. Accessibility of RPs and the familiarity of clinical environment and staff were also appreciated.

“for people with chronic disease it’s beneficial for them to be seen closer to home because it takes much of the medicalisation out of it and it’s reassuring for patients” (Hospital doctor2)

“It’s closer. They don’t have as far to travel. They don’t have as many issues around parking the car or arranging transport or having to get two buses or that kind of thing. In this area we quite lucky because you can get a bus direct to the hospital but even so it takes time. I think most patients like being in an environment they’re more familiar with” (GP 2).

“by having me out there, they have a face – one person who they know, and it’s not just about the disease. I’m part of their family a lot of the time really. I lot of my patients who live alone, who are widowed, if they come into hospital I’ll visit them. I’m often their only visitor. It’s the pastoral side of things as well. I’ll take the nighties home and I’ll wash them and bring them back to the hospital, and I’ll nip in and feed the cat and do things like that. So I think from that side of things the benefit’s there. And also I think the GPs benefit greatly because there’s not there’s not the drain on their limited resources because I’m there to deal with the Rheumatology patients because that can be quite big, especially if they’re flaring or the disease is uncontrolled” (RP 3)

### Perceived disadvantages for patients

Few disadvantages were identified. However, the most frequently identified disadvantage was lack of other onsite services (for example X-ray) requiring patients to travel to hospital for access to those services.

“Lack of facilities. You’ve got everything on tap in a hospital. They’re a bit isolated so there isn’t a doctor sitting round the corner to ask. A bit unsupported – with staff and equipment” (Hospital doctor 4)

“Most of the time I don’t think it is an issue but I suppose if patients were presenting acutely and needed investigations - needed a scan, needed an x-ray, they could get it done on site at the same time rather than having to revisit” (GP 1)

Some participants indicated the lack of back up medical expertise onsite to be a disadvantage.

“I suppose the disadvantages of a nurse running a clinic independently is that she hasn’t got Consultants that she can talk to although I know that the nurse who works here has a very good relationship with the GPs and she can always talk to the patient’s own GP about the patient” (Practice nurse 2)

### Types of patients

Most participants agreed that RPs should see stable patients who have had their initial diagnosis and treatment plan formulated in the hospital. Participants also agreed that RPs should see not only RA patients but certain other patients with chronic inflammatory arthritis (such as psoriatic arthritis, ankylosing spondylitis) as long as they are stable. Another point, which was raised by some participants, is the possibility of using the service as a triage service or a first contact assessment for rheumatology patients before hospital referral. While some RPs, GPs and nurses acknowledged that this does happen in their practices and that they do refer or accept referrals within their practice for initial opinion, others said that they could not refer to RPs directly and any RP referral has to come through rheumatology consultants. This part of the service seemed to vary among practices and there was neither consensus nor clarity whether RPs should provide this service or not.

“.. all of the different forms of arthritis. Patients with other connective tissue diseases – we have a lady with Wegener’s granulomatosis; we have people with Lupus who are seen in outreach clinics, so I think really the full spectrum of people who have a diagnosis and who have been settled and are reasonably controlled” (GP 2)

“Follow-ups are good. If it’s new patients my feeling is that new patients ought to be seen here but if we were being pressurised to see new patients in an outreach clinic setting, I would have thought soft tissue problems would be the sort of thing that could quite easily succeed in an outreach clinic” (Hospital doctor 2)

“there are almost no patients who can’t be seen in Outreach clinics. The question is whether patients should be seen only in Outreach clinics or virtually only in Outreach clinics and very rarely in hospital clinics or patients that should be combined care. I can’t think of any patient actually – with co-morbidities, with complex disease, that can’t be seen in an Outreach clinic and in hospital. What you do is reduce the number of hospital clinics. I suppose patients in whom there is a question mark about the diagnosis – it’s not appropriate if we’re still doing diagnostic tests, that’s not appropriate for a patient but most patients can be seen in Outreach clinics but some will have to be seen in hospital clinics as well” (Hospital doctor 1)

### Cost effectiveness

The service was viewed as being cost effective for patients as it saves them the financial cost of travelling to hospital, parking and waiting times. However, participants were not sure as to its cost effectiveness for staff and service providers. Some suggested that it may be cost-effective for service provider as most of the patients are old and their transport to hospitals is provided by the NHS. It was recognised that cost effectiveness requires considered analysis due to its complexity which needs to take into consideration patient satisfaction and quality of service provided.

“if you address someone’s concerns early on in the illness and educate them appropriately that’s bound to have an effect later on. If you teach them about the chronic illness they have and encourage them to manage their disease themselves and again that will have benefits” (Hospital doctor 3)

“you’d need a health economist to work out the cost-effectiveness properly. I mean, certainly for patients it’s better – it’s a lot cheaper – no parking, less travelling. If you look at it from an environmental point of view, you would have thought it would be a winner” (GP4)

“For patients they would love it but obviously if you are looking at the other costs, is it cost-effective? You would think it would be cost-effective but I suppose you can always cut costs in places” (Practice nurse 1)

### Primary/ secondary care communication (and benefits)

Most participants agreed that the service helped to bridge the communication gap between Primary and Secondary Care. GPs and nurses felt comfortable communicating with RPs if they have a query about a rheumatology patient. There were some variations, however, regarding knowledge transfer to Primary Care professionals. While some GPs and nurses praised the rheumatology knowledge and updates they received from their RPs, some seemed to think more could be done in this regard with more organised educational sessions conducted by the RPs in their practices.

“easier to ask the outreach practitioner a question than to try to speak to one of the Consultants or one of the registrars”. (GP3)

“Things happen quicker”. (RP 2)

“Outreach clinics improve communication between primary and secondary care rheumatology services” (Practice nurse 3)

“Communication between GP and practitioner better therefore communication between GP ad secondary care improved as RP an intermediary – this helps patients if there are complications” (GP 1)

### Training for health professionals

A perceived benefit for Primary Care was that, expertise normally confined to Secondary Care, was brought directly in to the community. Concerns were however raised by trainees in Secondary care about lack of training in routine care if these patients were all cared for in the community.

“Perhaps you might say that there’s a training effect: the junior doctors in clinics are missing out on that part of clinical practice so don’t get the same practice in monitoring disease-modifying drugs as they would if the patients were coming in but I think that’s a minor issue” (Hospital doctor 1)

## Discussion

This is the first study to explore the views of health professionals from different disciplines on the advantages and disadvantages of Primary Care based clinics for chronic disease. We studied Primary Care based rheumatology clinics in Norfolk because of the unique model of specialist nurse practitioners working in Secondary Care and general practice. The opinions of participants have suggested that this unique service model is valued by health professionals in both Primary Secondary Care, and there was a high degree of agreement across both sectors.

The development of care close to home is a key government policy. The policy is, however, controversial because of fears about fragmentation, loss of continuity of care and increased costs. The present study suggests that given an appropriate structure, a community based rheumatology service can be established which is satisfactory to both Primary and Secondary Care health professionals. Nevertheless, the study did highlight some areas of concern, particularly a lack of many investigative facilities in the community. However, routine phlebotomy is available in community practices, and the follow up of stable patients in the community rarely requires diagnostic radiological investigation. In addition most radiological investigations other than plain radiography require a specific visit to hospital. Thus for most patients there is a trade off between the convenience of community clinics for the occasional inconvenience of a trip to hospital which might have been avoided had they been seen initially on the hospital site.

Chronic diseases such as rheumatoid arthritis require long-term follow up to monitor the condition, co-morbidities and also response to therapies including toxicities. Much routine follow up can be performed by well trained nurse practitioners rather than physicians and many studies have shown equal outcomes [5,17,18].

The hub and spoke model described in this study is, we believe, is unique. Could our findings be applied in other geographical areas or specialities? We believe they could: many of the findings are not specific to our model, geographical area or RA. In particular there is a need for close integration.

### Limitations of the study

The qualitative design enabled us to explore in depth the opinions of staff across both Primary and Secondary care.

We only interviewed staff working in practices with an established rheumatology clinic. It is likely that as the clinic was well established, they would be more likely to view it in a favourable light. Practices without outreach clinics might view their development differently and we hope to explore this subsequently.

Due to technical reasons we did not have a complete set of transcripts from each outreach centre. However, researcher’s written notes from those interviews were used to complement the analysis. We do not have any reason to suppose that the centres used differ in any way from the others.

The sample size was small because we only interviewed staff at one secondary care hospital and seven general practices. This may limit the generalizability of our findings. However, many of the key aspects such as the importance of integration are generally applicable.

There have been several previous studies looking at the effectiveness of outreach clinics [15,19]. Patient satisfaction with physician outreach clinics is high [19]. The key reasons for this are: being seen closer to home, parking facilities and continuity of care. Interestingly in a patient survey study based in a suburban, industrialised area of Birmingham UK there was overwhelming desire from the patients to be seen in the hospital clinic and not in a Primary care setting with 99.3% of patients preferring the hospital site [20]. This was attributed to ready access to all facilities in the hospital and the short travelling distances. This study was based in an urban/rural environment only one practice was based within Norwich City (the others were based up to 45 kilometres from the hospital). Thus, travelling time is likely to be a major reason why patients preferred the community setting.

Gruen, in a review of outreach clinics conducted by physicians, concluded that whilst the patients benefitted from outreach clinics they were felt to be inefficient and inconvenient by physicians because fewer patients were seen per hour in outreach having allowed for travel time. [19]. However, in this study the outreach clinics are conducted by nurse practitioners not by doctors and are set up with a time template identical to that in Secondary care.

## Conclusions

Care closer to home is generally perceived as bringing significant benefits to the patient with improved outcomes together with cost savings. However, the evidence base for this assertion has not been rigorously tested. The present study is the qualitative component of a larger study designed to answer this question with a detailed analysis of the costs involved in providing rheumatology care in the community. We have analysed a service, which is closely linked across both Primary and Secondary care as the practitioners involved work across both sectors. This integration was highly valued by those interviewed. Rheumatoid arthritis has a prevalence of 0.8%. Therefore, a practice with 10,000 adults will have 80 patients with RA. Moreover, drug therapies for RA are becoming increasing complex with the advent of combination immunosuppressive therapies and biologic therapies. An individual practice may not, therefore, have the expertise to manage this specialist group of patients. Integration across both sectors enables skilled personnel to work in the community and achieve the policy desired care closer to home.

The restructuring of the health service with development of GP commissioning consortia is likely to lead to further development of community based services for chronic conditions. It is clear from this study that such services can work well but that the key is integration across Primary and Secondary care. Development of community services independent of specialist care will lead to service fragmentation and potentially deterioration in quality of care and increased cost. The costs of our model to both patient and the health economy are currently being directly evaluated, with a direct comparison of the costs of care in Primary versus Secondary care.

We therefore recommend that commissioners establishing community services consider developing a hub and spoke model with Secondary care as this will bring care closer to the community without losing benefits of access to a specialist centre of expertise. Patients with stable well controlled disease are most suitable for this approach, as they are the group of patients least likely to require Secondary care input. Guidelines and pathways for management of patients in the community should be developed to ensure that best practice is shared across all sites. The expressed opinions and justifications of the participants with respect to the benefits of RPs becoming qualified and approved prescribers should be considered.

## Competing interests

We have no conflicts of interest or competing interests to declare.

## Authors' contributions

RAW, JM, AJM, DGIS and GB were responsible for the study conception and design. AA conducted all interviews. AA, JM and AW performed the data analysis. AA, JM and RAW drafted the manuscript. All authors commented on the draft manuscript. All authors read and approved the final manuscript.

## Pre-publication history

The pre-publication history for this paper can be accessed here:

http://www.biomedcentral.com/1472-6963/12/119/prepub
